# Travel-Induced Circadian and Microbiota Disturbances: Implications for Athlete Health and Performance: A Narrative Review

**DOI:** 10.3390/nu18101523

**Published:** 2026-05-11

**Authors:** Karol Biliński, Kacper Wiśniewski, Laura Rafner, Paweł Witko, Dagmara Gaweł-Dąbrowska

**Affiliations:** 1 Faculty of Medicine, Wroclaw Medical University, Wybrzeże L. Pasteura 1, 50-367 Wroclaw, Poland; karol.bilinski@student.umw.edu.pl (K.B.); kacper.wisniewski@student.umw.edu.pl (K.W.); laura.rafner@student.umw.edu.pl (L.R.); pawel.witko@student.umw.edu.pl (P.W.); 2 Department of Population Research and Prevention of Civilization Diseases, Wroclaw Medical University, Chałubińskiego 3, 50-368 Wroclaw, Poland

**Keywords:** circadian misalignment, gut microbiota, jet lag, short-chain fatty acids, chrono-nutrition, skeletal muscle metabolism, athletic performance

## Abstract

High-performance athletes are increasingly exposed to frequent trans-meridian travel, leading to profound circadian desynchronization and gastrointestinal distress. This review examines the complex interplay between the host’s central circadian system and the gut microbiota (GM), both of which exhibit synchronised daily oscillations essential for homeostasis. Rapid time-zone transitions, such as those anticipated for the 2026 FIFA World Cup, induce a state of “gut jet lag,” characterised by the loss of rhythmic microbial functions and impaired intestinal barrier integrity. Circadian misalignment is associated with increased systemic inflammation and disrupted metabolic regulation, which may contribute to impairments in cognitive performance, sleep quality, and muscle recovery. Critically, travel-induced dysbiosis may reduce the production of microbial metabolites, specifically short-chain fatty acids (SCFAs) like acetate, propionate, and butyrate. These SCFAs serve as energy substrates that may enhance glucose uptake, lipid oxidation, and glycogen storage in skeletal muscle. Evidence suggests that travel-related stressors—including dehydration, psychological stress, and shifts toward highly processed diets—further exacerbate the loss of beneficial taxa. To mitigate these effects, this article proposes evidence-informed strategies: timed light exposure to reset the master clock, chronobiotic meal timing to entrain peripheral tissues, and targeted symbiotic supplementation to restore SCFA-producing populations. Integrating these personalised, evidence-informed protocols may support the optimisation of physiological resilience and performance.

## 1. Introduction

The circadian system is crucial for a wide array of physiological processes like sleep–wake cycles, hormone secretion, immune function, and energy metabolism. Central to this system is the suprachiasmatic nucleus (SCN) of the hypothalamus, which synchronises peripheral clocks across tissues and organs. Disruptions of this complex rhythm due to irregular sleep, transmeridian travel or night shifts can lead to desynchronization of the internal clocks, leading to impairment of metabolic regulation and increased systemic inflammation [1,2].

Simultaneously, the gut microbiota (GM) has recently emerged as a key regulator of host homeostasis. It contributes to nutrient absorption, immune modulation, and even neuroendocrine signalling. Intriguingly, growing evidence suggests that the GM also exhibits its own circadian rhythms, which are influenced by feeding behaviour, light cycles, and host circadian genes. This two-way communication has emerged as a key factor in maintaining overall health and well-being [3,4,5].

Athletes are highly susceptible to circadian cycle and GM disturbances. Frequent travel across time zones often causes jet lag. This, in turn, impairs cognitive performance, sleep quality, immune resilience, and muscle recovery. Beyond these direct effects, recent studies suggest that jet lag also induces shifts in GM composition, disturbing pathways essential to performance and recovery [6,7].

It is well established that training itself modulates GM, with elite athletes exhibiting characteristic microbial profiles. Elite athletes are shown to have increased GM diversity and the presence of taxa associated with improved energy metabolism [8]. However, when athletes are exposed to constant travel, it may mitigate the positive adaptations and compromise health and performance [9,10].

Athletes now travel more frequently than ever before and are therefore highly susceptible not only to jet lag but also to additional travel-related stressors, including dehydration, repeated dietary shifts, and psychological stress [11]. These factors collectively contribute to disruptions in GM composition and function. This issue is particularly timely given the upcoming 2026 FIFA World Cup in North America, which will bring athletes from across the globe into rapidly changing environmental and temporal contexts [12,13].

This narrative review aims to synthesise current evidence on how circadian disruption influences the GM and how these alterations, in turn, affect physiological processes critical for athletic performance. Despite increasing research, an integrated understanding of how circadian disruption interacts with gut microbiota in athletes exposed to travel-related stressors remains limited. This review addresses this gap by defining travel as a multifactorial exposure and by linking circadian misalignment with microbiota-related mechanisms affecting metabolic regulation, recovery, and performance.

## 2. Methodological Approach

This article was designed as a narrative review aimed at providing an integrative overview of current evidence on travel-related circadian disruption, gut microbiota, and athletic performance.

A targeted, non-systematic literature search was conducted using databases including PubMed, Scopus, and Web of Science to identify relevant studies. The search primarily focused on publications from 2016 to 2026, while earlier seminal studies were included to provide essential physiological context.

Relevant literature was identified using combinations of keywords such as “gut jet lag,” “short-chain fatty acids,” “circadian rhythms,” and “athletic recovery,” along with related metabolic and neuroendocrine terms. Both human and animal studies were considered where appropriate.

Studies were selected based on their relevance to the topic and their contribution to the conceptual framework of this review, with particular emphasis on circadian disruption, gut microbiota interactions, metabolic regulation, and exercise-related outcomes.

This narrative review does not follow a formal systematic review protocol; rather, it aims to synthesize and contextualise existing knowledge to highlight emerging mechanisms and potential applications in athletic settings.

## 3. Circadian Rhythms and Jet Lag in Athletes

### 3.1. Mechanism of the Circadian Cycle

Circadian timing has shaped human physiology and behaviour for millions of years. A large share of metabolic and physiological processes exhibit daily oscillations under clock control [1]. In mammals, the primary circadian regulator is situated within the SCN of the anterior hypothalamus. The initial empirical observations supporting its crucial function emerged in the early 1970s, when ablations of the SCN in rodents resulted in a total disruption of circadian rhythm in both locomotor behaviour and endocrine processes [14]. Two decades later, SCN tissue grafts restored rhythmicity and imposed the donor period in arrhythmic hamsters whose SCN had been ablated. Taken together, these classic studies established the SCN as the necessary and sufficient master pacemaker of the mammalian circadian system [15]. Each neuron contains a cell-autonomous clock built from transcription–translation feedback loops (TTFLs): CLOCK/BMAL1 activate Per1/Per2 and Cry1/Cry2, and accumulating PER/CRY complexes repress CLOCK: BMAL1-driven transcription. Nevertheless, synchronisation of these neurons is essential for the SCN to generate a coherent and stable output rhythm for humans, requiring network coupling via neurotransmitters and neuropeptides such as vaso-active intestinal peptide (VIP), γ-aminobutyric acid (GABA), and arginine vasopressin (AVP), for which VIP → VPAC2 signalling is pivotal for synchrony [16,17,18,19].

At the systems level, photic input aligns these cellular oscillators with the external environment. Light affects SCN via the retinohypothalamic tract, which is formed by intrinsically photosensitive retinal ganglion cells (ipRGCs). This input sets the phase of the SCN clock relative to the light–dark cycle. The human phase response curve (PRC) to light is referenced to the core body temperature minimum (CBTmin) [20]. Through autonomic and neuroendocrine pathways, the SCN regulates cortisol via pituitary ACTH release and modulates adrenal sensitivity through the autonomic nervous system. It also drives pineal melatonin via sympathetic output and shapes sympathetic–parasympathetic balance, influencing insulin sensitivity, hepatic glucose production, cardiovascular tone, and pancreatic insulin release [21].

### 3.2. Circadian Disruption Induced by Trans-Meridian Travel: Mechanisms and Physiological Consequences

#### 3.2.1. The Influence of Light and Travel Direction on Circadian Desynchrony

Rapid time-zone transitions produce jet lag, which is a phase misalignment between the SCN and local time caused by a rapid time zone shift. This misalignment causes characteristic jet lag symptoms, including disrupted sleep (insomnia or frequent awakenings), daytime fatigue, diminished alertness, loss of appetite, gastrointestinal upset, and impaired physical and cognitive performance until the internal clock resynchronizes with local time. The timing of light exposure determines the direction of phase resetting: evening/biological-night light produces phase delays, whereas early-morning light produces phase advances (human PRC referenced to the individual’s CBTmin) [22].

However, even brief light exposure is sufficient to shift the clock appreciably. For example, a single 1 h pulse of ~8000 lux bright light can induce a phase delay of about 2 h and a phase advance of about 1.2 h.

Short-wavelength light (~460–480 nm) is most potent at suppressing melatonin, reinforcing the risk of evening blue-enriched exposure during travel. This effect is mediated by melanopsin-expressing ipRGCs projecting to the SCN [20].

Humans’ intrinsic period is slightly longer than 24 h (~24.18) h, which explains the asymmetry: westward travel (phase delays) is easier to adapt to than eastward (phase advances), with a typical re-entrainment rate of ~1.5 h/day west versus ~1 h/day east [23]. In elite track cyclists, long-haul travel, particularly eastward, considerably diminishes sleep duration (time in bed and total sleep time) and adversely affects sleep efficiency for approximately 48 h following arrival, resulting in greater fatigue at bedtime. Actigraphy data demonstrated pronounced reductions in time in bed, total sleep time, and sleep efficiency during this 48 h post-travel window, after which these measures began to return toward baseline. Complementary sleep diary data further indicated significant reductions in sleep quality and increases in pre-sleep fatigue associated with long-haul eastward travel. The extent of sleep disruption observed in this period may negatively affect both the athlete’s health and performance [24]. Similar results were observed in female and male soccer players travelling east from Ireland to Taiwan. In this study, Biggins et al. monitored subjective sleep and well-being measures, and actigraphy was also performed. It was concluded that sleep remained negatively impacted for up to five days. It is also noteworthy that female players reported greater pre-sleep tension and anxiety compared with male players at all time points. Despite these emerging conclusions, it remains unknown whether these effects interfere with training and performance [25]. However, while subjective impacts are clear, objective performance links in other professional sports are further explored in Section 3.4.

#### 3.2.2. Non-Photic Influences on Circadian Timing: Exercise, Diet, and Social Cues

There are also significant factors beyond light that can cause phase shifting. Exercise has been shown to shift circadian phase in both animal models and humans [26,27]; depending on its timing, exercise can induce either phase advances or phase delays of the clock, with human phase-response curves indicating advances after morning and early-afternoon sessions and delays after late-evening exercise. Recent evidence further demonstrates that exercise produces significant phase shifts in multiple melatonin rhythm markers, particularly in the onset of aMT6s excretion, a robust phase indicator, while also inducing changes in melatonin duration driven primarily by onset shifts [26]. Importantly, these phase-shifting effects were consistent among young and older adults and between sexes, and the observed morning and evening delay/advance regions closely resembled those of bright-light PRCs. Exercise performed during the early- and mid-afternoon elicited a particularly robust phase-advancing effect, indicating that physical activity within this temporal window may exert a stronger influence on circadian timing than traditionally appreciated [26]. These findings suggest that afternoon exercise may serve as an effective zeitgeber—an external cue that entrains or shifts the circadian clock—particularly for individuals who are unable to exercise earlier in the day. In a study involving 52 young adults, morning exercise was found to induce significantly greater phase-advancing shifts compared with evening exercise [28]. The investigators also observed that chronotype modulated the circadian response to timed physical activity. Individuals with later chronotypes exhibited phase advances following both morning and evening exercise, whereas those with earlier chronotypes displayed phase advances in response to morning exercise but phase delays after evening exercise. These findings underscore the importance of personalised exercise timing in optimising circadian alignment. In addition to physical activity, social interactions and feeding schedules also function as potent non-photic zeitgebers capable of modulating circadian phase. Feeding schedules primarily adjust peripheral circadian clocks (e.g., in the liver and gastrointestinal tract) [29]. For example, restricting food intake to certain times of day can shift the liver’s clock gene expression even when the central SCN clock is held constant. Evening caffeine consumed around 3–4 h before habitual bedtime has been shown to delay the melatonin rise by roughly 40 min [30].

#### 3.2.3. Hormonal and Metabolic Consequences of Circadian Disruption

Hormonal axes also exhibit dysregulation under conditions of repeated circadian disruption. One study reported that flight attendants repeatedly exposed to jet lag (i.e., long-haul crews) demonstrated significantly elevated daytime cortisol levels and impaired cognitive performance, such as slower reaction times on attention-based tasks, compared to a ground crew control group. These differences were observed only on long-haul flights, but were not observed after domestic flights [31]. Eastward travellers (clinical sampling) exhibit phase-shifted cortisol circadian rhythms, characterised by heightened late-night cortisol levels and shifted acrophase for at least 36 h post-return—suggesting post-flight hypothalamic–pituitary–adrenal (HPA) axis misalignment [32]. In relation to mechanism, the adrenal gland’s local circadian clock (which is BMAL1-dependent) modulates the timing of glucocorticoid peaks, providing a basis for transient SCN–adrenal desynchrony after travel [33]. Studies suggest that disruption of the circadian rhythm can impair glycaemic control [34,35]. Controlled forced-desynchrony work shows that misalignment raises levels of glucose and insulin and lowers leptin, with certain postprandial glucose responses characteristic of a prediabetic state, thereby illustrating that circadian misalignment engenders detrimental metabolic consequences pertinent to jet lag [35].

### 3.3. Consequences for Metabolism, Immune Response, Recovery, Stress, Cognitive Function, and Inflammation

Circadian misalignment in shift workers carries clear metabolic and cardiovascular costs. In a tightly controlled study (with sleep and meals cycled through all phases), circadian misalignment produced a ~17% decrease in leptin, ~22% increase in 24 h insulin, and ~6% higher 24 h glucose, due mainly to exaggerated post-prandial spikes (some readings entered the prediabetic range). At the same time, the normal cortisol rhythm inverted: cortisol was abnormally low upon waking and elevated toward the end of the wake period—an endocrine profile predisposing to insulin resistance. Mean arterial pressure also rose by ~3% (~3 mmHg), and sleep quality deteriorated, with sleep efficiency dropping by ~20% (e.g., from ~84% to ~67%). Taken together, these findings suggest a transient cardiometabolic stress state during re-entrainment, which may influence short-term performance and potentially contribute to longer-term risk [35].

Evidence from human and animal studies shows that circadian disruption may affect both innate and adaptive immunity and shifts the timing of immune activity. In animal models, chronic circadian misalignment has been associated with increased stimulus-driven IL-6 responsiveness, primarily due to altered immune cell reactivity rather than changes in cell number [36]. In humans, being on a night-shift schedule can desynchronize the usual coordination between immune cell counts and cytokine release [37]. Specifically, the peak of cytokine responsiveness may become uncoupled from the normal daily rhythm of leukocyte numbers; concurrently, rotating/night-shift workers show altered circulating cytokine, chemokine, and growth-factor profiles, with many analytes lower at night than by day, suggesting alterations in immune pathways and potential reductions in vaccine responsiveness and infection control [37,38].

### 3.4. Circadian Cycle Disturbance in Sport: Reaction Time, Endurance

Disruptions in circadian rhythms may influence athletes’ reaction times and recovery capacity, as these biological processes regulate key physiological and psychological functions required for performance [39]. They not only elicit a spectrum of unpleasant general symptoms, including physiological dysregulation, sleep disturbances, fatigue, diminished appetite, and gastrointestinal anomalies, but may also be associated with impairments in both physical and cognitive performance, displacing athletes from their ideal temporal performance window and diminishing overall efficiency [40].

Individuals also have different chronotypes, some tending toward “morning-lark” (early) or “night-owl” (late) profiles. A good deal of consistency was observed across studies, with nine genes identified in two of the three genome-wide association studies, and several genes previously unknown to influence chronotype were also identified [41]. Chronotype has been shown to influence both cognitive and physical performance. In the test of Maximal Voluntary Contraction (MVC) at 08:00 h, ECTs (early chronotypes) performed 7.4% better than LCTs (late chronotypes) in MVC, whereas at 20:00 h, LCTs performed 3.7% better than ECTs. For ECTs, cognitive performance is best almost immediately after wake-up, and physical performance (like MVC) peaks between 5 and 7 h after wake-up. In contrast, LCTs do not reach their peak performance until at least ≥12 h after entrained wake-up time [42]. Even small differences may be relevant at the elite level. A study measuring Olympic swim performance found that the fastest swim times occurred around 17:12 h, indicating a ~0.32% improvement in performance compared to 08:00. Notably, this effect exceeded the typical performance differences between medal positions. Notably, the magnitude of this circadian variation exceeded the time differences between medal positions in a substantial proportion of races, including the time gap between gold and silver in 40% of races, between silver and bronze in 64%, and between bronze and fourth place in 61% of finals [43].

Consistent with this, the direction of travel plays a pivotal role. Eastward travel has been linked to impaired performance and game outcomes in the NBA. Teams playing with eastward jet lag (e.g., a West Coast team playing on the East Coast shortly after travel) saw their win percentage drop by about 6%. Jet-lagged teams also showed worse shooting accuracy and a poorer point differential compared to their normal performance. In that study, a 2 h circadian misalignment (≈two time zones) corresponded to the jet-lagged team scoring approximately 4–5 fewer points on average [44].

A study on Major League Baseball (MLB) performance also found jet lag effects, primarily after eastward travel. Eastward travel was associated with declines in home-team offensive performance (e.g., fewer runs scored), whereas westward travel had very limited effects. Interestingly, away teams were not similarly affected, suggesting the performance impairment was specifically due to the home team’s jet lag [45].

Timing of assessment is also critical: negative effects of jet lag are most prominent in the first 24–72 h after travel (particularly after eastward flights), a pattern consistent with long-haul Super Rugby findings. In that 11-year analysis, teams undertaking approximately 24 h of eastward long-haul travel across 12 time zones showed clear, statistically meaningful reductions in multiple key performance indicators—even after accounting for secular trends, rule changes, competition format shifts, team ranking, and the away-match disadvantage—suggesting that the duration and magnitude of circadian disruption may influence match performance [10].

## 4. Gut Microbiota and Circadian Regulation

Circadian rhythms and the GM are closely interconnected. The gut microbial community is highly dynamic, exhibiting daily oscillations in composition and function, the phase and amplitude of which are largely influenced by the host’s feeding–fasting cycles. Timed meals can entrain peripheral clocks independently of the suprachiasmatic nucleus (SCN), thereby making meal timing a zeitgeber for intestinal microbes [46].

The SCN coordinates sleep–wake cycles that shape behaviours such as activity and food intake, which thereby modulate digestive motility, bile secretion, and intestinal metabolism—defining the temporal niche for gut bacterial oscillations [46,47]. The intestinal epithelial molecular clock (through the BMAL1/CLOCK transcriptional loop) also plays important role in generating temporal niches for microbes. Ablation of BMAL1 in intestinal epithelial cells has been shown to disrupt faecal microbial rhythmicity, alters the profiles of microbial metabolites (including branched-chain fatty acids and secondary bile acids), and transmits the dysregulated characteristics of the microbiome to germ-free animals. The peripheral intestinal molecular clock also partially autonomously controls intestinal IgA secretion, the properties of the intestinal mucus layer and gastrointestinal motility [48,49]. When these everyday phases are misaligned (e.g., jet lag, shift work), circadian misalignment has been shown to induce microbiota dysbiosis in animal models and has been associated with similar alterations in humans, which is associated with increased susceptibility to metabolic disorders and obesity [46]. The microbiota may also influence host metabolism and contribute to rhythmic changes in epigenetic regulation. It affects the expression and rhythmic chromatin recruitment of HDAC3 (histone deacetylase 3) in intestinal epithelium. HDAC3 rhythmically deacetylates histones and co-activates transcription factors (for example, ERRα), thereby contributing to microbiota-dependent diurnal metabolic gene expression such as that of CD36 (a key protein in lipid metabolism and the disposal of oxidised low-density lipoprotein) [50]. Bile acid pools exhibit circadian oscillations driven by feeding cycles and microbial bile salt hydrolase activity. Bile salt hydrolases produced by the intestinal microbiota convert conjugated bile salts into unconjugated bile acids, which act as signalling molecules that modulate the expression of circadian genes (e.g., *Dbp*, *Per2*, *Per3*, *Cry2*) in both the intestinal epithelium and the liver, as demonstrated following oral administration. Together, these findings suggest a signalling pathway linking the microbiota, bile acid metabolism, and circadian clock regulation [51]. In mouse models, intestinal clock disruption impairs immune cell recruitment and exaggerates colitis [52].

Empirical evidence derived from human observational studies and minor interventional research indicates that disturbances in occupational or behavioural circadian rhythms correlate with diminished microbial diversity and an increase in pro-inflammatory taxa. Recent work indicates that circadian rhythm disturbance (such as in shift workers) leads to changes in microbiota, particularly an increase in Muribaculaceae and a decrease in *Akkermansia*, which have been associated with compromised mucus barrier integrity and impaired barrier function, leading to intestinal inflammation and dysfunction [53]. In a randomised controlled trial involving 99 adults from India who reported sleep disturbances along with symptoms of depression and anxiety, circadian dysregulation was found to be associated with GM misalignment. Metagenomic analyses revealed a decrease in *Bacteroides* and a corresponding increase in *Firmicutes*. Furthermore, a significant reduction was observed in beneficial genera, including *Lactobacillus* and *Bifidobacterium* [54]. Social jet lag, characterised by misalignment between internal circadian rhythms and socially imposed schedules, has been associated with reduced production of short-chain fatty acids (SCFAs), which play a role in maintaining gut barrier integrity, host metabolism, and immune function, and may contribute to systemic inflammation [55]. However, much of the mechanistic evidence described above is derived from experimental and animal studies, and its direct relevance to elite athletic populations remains to be established.

## 5. Stress and Environmental Factors During Travel and Their Influence on Microbiota

### 5.1. Internal and Physiological Drivers of Microbiota Disruption During Travel

#### 5.1.1. Sleep Disruption and Circadian Desynchronization

Athlete health and performance are influenced by multiple factors, among which psychological and behavioural components are considered the most critical—including sleep, nutrition, hydration, recovery, stress, and the training environment [56,57,58,59]. Travel—particularly across distinct time zones, between continents, or into climates that differ markedly from one’s habitual environment—has been associated with adverse effects on physiological health. Emerging human evidence suggests that the GM may also play a role in these processes. For example, a recent proof-of-concept study in elite athletes reported that a multi-strain *Lactobacillus* intervention was associated with improvements in self-reported sleep quality (up to 69%) and energy levels (31%), alongside favourable changes in recovery-related hormonal markers [60]. However, these findings are based on small cohorts and should be interpreted with caution.

Beyond athlete-specific evidence, studies conducted primarily in non-athletic populations—particularly among frequent travellers and military personnel—suggest that jet lag may influence the composition and functional state of the GM. This impact has been reported to be age-independent and appears to be more pronounced at the species level than in the overall microbial composition [61]. The GM may exhibit its own circadian rhythm, synchronised with the host’s biological clock, which has been linked to intestinal health. Recent studies have proposed the concept of ‘gut jet lag’ to describe a state of desynchronization between the host’s circadian rhythm and that of the GM [7]. It has been suggested, primarily based on experimental and non-athlete studies, that gut dysfunction associated with “gut jet lag” may involve several mechanisms [62,63]. This may contribute to altered signalling and impaired propulsive contractions, which have been associated with slowed colonic transit and constipation. Gut jet lag has also been associated with impaired intestinal barrier function, which may increase the translocation of bacterial components such as lipopolysaccharides (LPSs) and may contribute to low-grade inflammation. This inflammatory state has been hypothesized to influence neuromuscular function, although direct evidence in athletes remains limited [7].

Several key factors influence sleep efficiency, including sleep duration, sleep regularity, the quality of the sleep environment (noise, light, temperature, and bed comfort), psychological state, level of physical activity, and dietary habits [64,65,66]. According to available human studies, GM diversity—including the abundance of bacteria from the Bacteroidetes and Firmicutes phyla—is positively correlated with higher sleep efficiency and total sleep duration [67]. Importantly, most of the described mechanisms are derived from non-athlete populations or experimental models, and their direct relevance to elite athletic performance remains to be established.

#### 5.1.2. Neuroendocrine Stress Responses and Microbiota Alterations

Another important factor influencing GM is the HPA axis and the Sympathetic Nervous System (SNS). A bidirectional relationship between GM and the HPA axis has been observed. It has been suggested that the GM may modulate the HPA axis through multiple pathways, including SCFAs [68,69,70], bile acids [71,72], Branched-Chain Amino Acids (BCAAs [73]), neurotransmitters (serotonin, GABA, dopamine, noradrenaline, neuropeptide Y, vasopressin, and oxytocin), and the immune system (IL-1β, TLR receptors), while the HPA axis may modulate cortisol, which represents the central focus of most research conducted in this field [73]. Among the most important intestinal cells susceptible to the effects of cortisol are epithelial cells, goblet cells, Paneth cells, immune cells (including lymphocytes, mast cells, and macrophages), and enteroendocrine cells [73,74].

Evidence suggests that cortisol may, among other effects, alter intestinal motility and mucus production, which may contribute to changes in the composition of human GM in favour of dysbiosis [75]. Alterations in intestinal transit time, increased permeability—primarily due to the loosening of tight junctions [76,77]—and changes in nutrient availability may collectively modify the gut environment. Critical proteins regulating intestinal barrier integrity comprise occludin, claudins, zonula occludens (ZO-1/2/3), junctional adhesion molecules (JAMs), and tricellulin [78,79]. Available data suggest that GM composition may be both directly and indirectly influenced by the integrity of the intestinal barrier [80,81]. Physical activity is another factor that may influence intestinal barrier permeability. Both human studies in athletes (e.g., endurance runners) and animal models suggest that intense exercise may increase intestinal permeability, potentially through mechanisms involving hypoxia and heat stress [78]. Moreover, a decrease in beneficial species, such as *Faecalibacterium prausnitzii*, and an increase in potentially pro-inflammatory species have also been reported [82]. However, many of these mechanisms are derived from experimental and non-athlete studies, and their relative contribution in real-world athletic settings remains unclear.

These mechanisms may be particularly relevant in athletes exposed to high training loads and competition-related stress, where HPA axis activation, gastrointestinal disturbances, and performance outcomes are closely interconnected.

#### 5.1.3. Dehydration and Intestinal Hypoperfusion

In athletes, hydration status is a key determinant of physiological function during training and competition. Emerging evidence also suggests that it may influence GM status by supporting intestinal perfusion, enhancing SCFA production when combined with prebiotics, modulating infection risk, and regulating intestinal barrier permeability [83]. Dehydration, oxidative stress, and overheating commonly accompany sports competitions and intensive training. These factors, observed also in professional soldiers [84] (often used as a model of prolonged physical stress comparable to endurance athletes), may promote intestinal hypoperfusion, which may increase intestinal barrier permeability and contribute to dysbiosis. Dysbiosis has been associated with gastrointestinal disturbances such as nausea, vomiting, diarrhoea, and abdominal pain, a set of symptoms commonly observed among endurance athletes [83]. Further research requires standardised sampling approaches, as hydration status interferes with bowel movement frequency, stool consistency, intestinal transit, and the living environment of GM. Consequently, this variability prevents the drawing of definitive conclusions [85]. Studies in intensively trained soldiers suggest that individuals with enhanced endurance may exhibit a higher abundance of bacteria capable of tolerating oxidative stress and producing SCFAs. Additionally, butyrate and propionate have been shown to facilitate the absorption of water and sodium ions from the intestinal lumen, thereby possibly contributing to improved metabolic adaptation [84]. These interactions may be particularly relevant in endurance athletes, where dehydration, thermal stress, and gastrointestinal symptoms frequently co-occur during prolonged exercise and competition.

### 5.2. Environmental and Behavioural Modulators of Microbiota Composition

#### 5.2.1. Dietary Patterns and Nutritional Challenges During Travel

Research indicates that travel and environmental changes are highly likely to disrupt adherence to dietary recommendations, which may affect health over the long term [86]. Dietary tendencies during travel are marked by increased intake of highly processed and fast foods, largely due to limited kitchen access and the need for quick meals. There is also a reduction in fruit and vegetable intake, connected with difficulties in accessing fresh produce while travelling. An increase in alcohol and sugar-sweetened beverage intake is also observed, commonly linked to both leisure and business travel, and often perceived as part of the travel experience. Additionally, travel has been linked to disruptions in meal timing, with food intake occurring at atypical hours due to jet lag and time-zone shifts inherent to long-distance travel. Finally, changes in hydration status are frequently observed, with the potential to further affect dietary balance and overall health [87].

Diet, due to its direct action on the intestines, is mentioned as probably one of the most significant influences on the state of the GM. Dietary modifications, including elevated consumption of proteins, fats, or dietary fibre, may be mirrored in the composition and functional state of the GM, which could potentially exhibit dynamic fluctuations within just a few days [84,88].

Certain professional groups characterised by frequent travel may provide valuable insights into the potential impact of travel—particularly air travel—on GM composition. Airline pilots represent one such group. In a 2023 study conducted by Minoretti et al., GM profiles in pilots exhibited lower abundances of several beneficial taxa, including *Akkermansia muciniphila* (~31% lower), *Lactobacillus* spp. (~15% lower), and *Faecalibacterium prausnitzii* (~8–10% lower), compared with fitness instructors [89].

Both *Akkermansia muciniphila* and *Faecalibacterium prausnitzii* are regarded as beneficial gut bacteria that are routinely associated with supporting microbiota health. *Akkermansia*, through the degradation of mucin, has been associated with the regulation of the intestinal mucus layer and metabolic processes, and may facilitate communication between the microbiota and the immune system. *Faecalibacterium*, in turn, is associated with producing SCFAs, including butyrate. A reduced abundance of these taxa has been associated with dysbiosis, impaired barrier function, and pro-inflammatory states, which may potentially be correlated with metabolic and immune-mediated disorders [90,91,92].

Another study from 2023, conducted by Worby et al., analysed stool samples from 267 non-athletic individuals in the United States who undertook international travel between 2017 and 2019 [93]. Samples were collected before departure and after return to assess changes in GM composition. The cohort consisted of 60% women and 40% men, with travel destinations including South America (18%), South Asia (16%), Southeast Asia (15%), and East Africa (15%). Traveller’s diarrhoea occurred in 33% of participants, and 12% reported antibiotic use during travel. Metagenomic analysis indicated that a reduction in GM diversity was observed in 61% of participants. Notable changes within the Enterobacteriaceae family included:

*E. coli*: median relative abundance increased from 0.1% to 0.6% (*p* < 1 × 10^−10^).

*Klebsiella* spp.: 33% of participants were found to have acquired new strains (vs. 8% lost; *p* < 1 × 10^−10^).

*Shigella* spp.: 26% of participants were found to have acquired new strains (vs. 1% lost; *p* < 1 × 10^−10^).

These findings highlight substantial microbiota alterations associated with international travel in non-athlete populations.

A decline in *Alistipes* spp. was observed, along with reductions in *Faecalibacterium, Bifidobacterium*, and *Ruminococcus* among individuals who used antibiotics during travel. A reduced abundance of these taxa has been associated with reduced SCFA production and alterations in epithelial barrier integrity, possibly contributing to a higher probability of increasing susceptibility to inflammatory and metabolic disorders [94,95,96]. Overall, the study demonstrated statistically significant alterations in GM following international travel, with particularly pronounced changes among non-athlete travellers to South Asia. These changes were associated with a loss of microbial diversity and the acquisition of potentially pathogenic Enterobacteriaceae strains. Intestinal colonisation with Enterobacteriaceae strains during travel may be connected with the resistance gene load, potentially increasing the risk of AMR transmission globally. Travellers, as vectors, may spread AMR (e.g., ESBLs), threatening public health. Furthermore, the decline in diversity and the growth of Enterobacteriaceae seem to exhibit a strong association with weakening the microbiota barrier, possibly facilitating pathogen colonisation [93]. It should be noted that this study did not account for whether participants were professional athletes, focusing solely on travel history. Although this study did not include athletes, these findings may have potential relevance for athletic populations, who are frequently exposed to international travel and similar environmental stressors.

#### 5.2.2. Sanitation, Microbial Exposure, and Antibiotic Risk

Hygiene, access to clean water, and sanitary conditions during travel or field training have been associated with changes in GM status [84]. Access to properly functioning sanitation facilities (e.g., toilets and sewage systems) has been associated with reduced exposure to faecal matter and bacteria carrying antimicrobial resistance genes (ARGs). During travel, increased exposure to contaminated environments may elevate the risk of GM colonisation by ARG-harbouring strains. Adequate sanitary conditions have been associated with a reduced likelihood of colonisation by resistant strains, which may otherwise influence GM composition, as reported in non-athlete studies [97]. Interestingly, recent studies suggest that increased hygiene levels in urban environments, including access to clean water and limited contact with environmental microorganisms, may also have unintended consequences. Such conditions have been associated with reduced GM diversity and stability, potentially due to restricted horizontal microbial transmission. Conversely, poor hygiene conditions may increase the risk of infections, often followed by antibiotic use, which can also reduce microbial diversity. Therefore, both excessive and insufficient microbial exposure may influence GM composition through different mechanisms. However, evidence in professional athletes remains limited, and the extent to which these factors influence performance-related outcomes is currently unclear.

## 6. Microbiota-Mediated Impacts on Health and Performance

Long-distance travel may contribute to GM dysregulation and circadian misalignment. The influence of the GM on athletic performance represents a novel research direction that has emerged in recent years. Available evidence suggests a bidirectional relationship between GM and exercise performance, with microbial activity affecting physical capacity and training influencing microbial diversity [98,99,100].

### 6.1. Microbiota Influence on Athletic Performance

Some evidence suggests that probiotic supplementation may influence skeletal muscle mass and exercise capacity in both humans and animal models. In murine models, GM depletion can be associated with muscle atrophy and reduced performance, while microbial reconstitution partially restores these parameters [99,101,102,103,104]. Collectively, these findings may indicate a potential role of the GM in modulating athletic performance. However, evidence in humans remains limited, and most data derive from animal studies.

The results of a randomised, double-blind, placebo-controlled trial investigating the possible effects of GM and probiotic supplementation on athletic performance and post-exercise recovery were presented in “The Impact of Gut Microbiome Modulation on Athletic Performance and Post-Exercise Recovery in Endurance Runners” [104]. The study enrolled 40 male long-distance runners. The intervention group (*n* = 20) received a probiotic formulation containing *Lactobacillus acidophilus*, *Bifidobacterium lactis*, and *Lactobacillus plantarum*. Compared with placebo, this group showed an increase in VO_2_max (4.7%) and time to exhaustion (7.2%). Post-exercise recovery was assessed using delayed onset muscle soreness (DOMS) and serum creatine kinase (CK) levels. The probiotic group demonstrated an 18% lower peak CK concentration and a 23% reduction in DOMS relative to placebo. These findings suggest that probiotic supplementation with the indicated strains may be associated with improvements in selected performance and recovery markers.

Lahiri et al. report that mice deprived of GM, compared to conventionally colonised mice, exhibited reduced muscle mass, a lower number of muscle fibres, and decreased expression of myosin heavy chains [102]. Moreover, an increased expression of Atrogin-1 and Murf-1, both of which are mediators of muscle atrophy, was observed. Transplantation of microbiota from unmodified donor mice reversed these alterations, potentially indicating a role of the GM in skeletal muscle integrity and function [102].

Nay et al. reported similar findings. Additionally, the authors examined the impact on muscle glycogen availability. Germ-free mice exhibited lower muscle glycogen levels, impaired glucose storage, and reduced glycogen availability, which were restored following gut re-colonization. These findings may indicate a role of the GM in regulating muscle energy metabolism [101]. Importantly, a substantial proportion of the mechanistic evidence described above is derived from animal models, and its direct applicability to elite athletic performance in humans remains to be established.

### 6.2. Exercise as a Microbiota Modulator: Bidirectional Benefits

Several studies have investigated the relationship between physical activity, independent of dietary patterns, and GM composition [100,105,106,107,108,109]. A possible association has been observed between exercise and an increased abundance of short-chain fatty acid-producing bacteria.

Martin et al. investigated a cohort of 50 young men with normal body weight and comparable dietary habits, stratified by aerobic capacity [110]. The cohort included both elite athletes (cyclists and football players) and non-athletes. In individuals with very high aerobic capacity (predominantly cyclists), the GM was characterised by a higher relative abundance of the genus *Prevotella*, particularly *Prevotella copri*. These bacteria are recognised producers of SCFAs, which have been associated with metabolic functions that may be relevant to exercise performance [111]. In contrast, individuals with lower aerobic capacity exhibited GM dominated by *Bacteroides* species (*Bacteroides uniformis*, *Bacteroides vulgatus*) and *Faecalibacterium prausnitzii*. Notably, subjects with very high aerobic capacity demonstrated reduced microbial diversity but a specialisation towards SCFA-producing taxa (*Prevotella copri* and *Phascolarctobacterium succinatutens*). Conversely, individuals with lower aerobic capacity displayed a more diverse microbial community in terms of species richness [111]. However, these observations are based on cross-sectional data and do not establish a causal relationship between microbiota composition and exercise performance.

### 6.3. Molecular Mechanisms: How Microbial Metabolites Enhance Muscle Function

The mechanisms presented in this section provide plausible explanations for potential GM-related influences on athletic performance; however, the available evidence is largely derived from animal studies. Direct data from athletes remain limited, and the conclusions should therefore be interpreted with caution. The potential beneficial effects of the GM on physical performance may be mediated, at least in part, by SCFAs, which may act through multiple pathways, including modulation of glucose and lipid metabolism in muscle, regulation of inflammation and muscle regeneration, effects on mitochondrial function in skeletal muscle, and enhanced resistance to oxidative stress. However, these mechanisms are predominantly supported by animal studies, and their relevance in humans is not yet fully established [112].

SCFAs produced by GM, particularly acetate, can be taken up by skeletal muscle and utilised as an energy substrate [113]. Germ-free mice exhibit reduced SCFA production and decreased treadmill endurance, while acetate infusion has been shown to reverse antibiotic-induced reductions in performance [114]. SCFAs influence glucose metabolism in muscle. In rat muscle cell lines, acetate has been shown to activate AMP-activated protein kinase (AMPK) [115]. AMPK regulates glucose uptake, fatty acid oxidation, mitochondrial biogenesis, and overall energy homeostasis in muscle. Its activation increases GLUT4-mediated glucose transport and enhances β-oxidation [116]. These effects may increase intracellular glucose availability and improve its oxidative utilisation, potentially contributing to enhanced skeletal muscle function and increased endurance [116].

The microbiota, through SCFAs, could also affect glycogen metabolism. Germ-free mice show reduced glycogen and ATP levels in muscle, as well as decreased mRNA expression of glycogen synthase [117]. In another study, transplantation of microbiota rich in SCFA-producing bacteria from highly endurance-trained athletes into mice was associated with increased muscle glycogen stores and improved insulin sensitivity [110]. SCFAs may contribute to the regulation of skeletal muscle responses to insulin. In germ-free mice, insulin administration failed to induce phosphorylation of Akt, a key kinase in the insulin signalling pathway [117]. Akt (PKB) phosphorylation is crucial for insulin-stimulated GLUT4 translocation, activation of glycogen synthase through inhibition of GSK3, and energy storage in the form of glycogen. Impaired Akt activation limits glucose uptake, reduces glycogen and ATP stores, and may thereby impair muscle performance [118]. Dietary butyrate supplementation in obese mice reduced fasting insulin levels by 50% and was associated with improved insulin responsiveness in an intraperitoneal insulin tolerance test compared with controls. Enhanced Akt phosphorylation was also observed [119].

The same study assessed the impact of SCFAs on lipid metabolism and mitochondrial function in skeletal muscle. In obese mice, dietary butyrate increased oxidation of isotopically labelled palmitate by 200% relative to controls. Expression of the nuclear receptor PPAR-δ, which promotes fatty acid oxidation in skeletal muscle, was also elevated. Increased nighttime oxygen consumption further indicated enhanced lipid oxidation. Serum cholesterol and triglyceride levels were reduced in butyrate-supplemented mice [119].

The study also evaluated possible effects of butyrate on muscle fibre composition, revealing a higher proportion of type I (oxidative, slow-twitch) fibres, which are rich in mitochondria, consistent with enhanced lipid oxidation and potentially improved aerobic capacity in animal models. This effect was absent in germ-free mice, further supporting the role of the microbiota in these adaptations.

**Figure 1:**
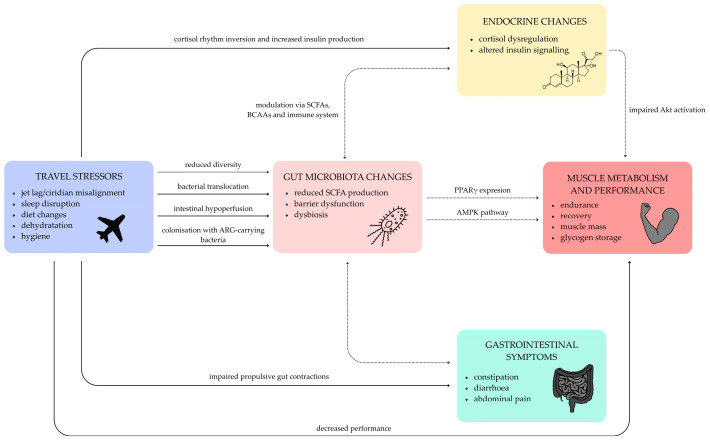
Conceptual model of interactions between travel-related stressors, gut microbiota alterations, and physiological responses relevant to athletic performance. Travel-related factors may influence gut microbiota composition and function, which in turn may interact with endocrine signalling, skeletal muscle metabolism, and gastrointestinal function. These interactions may contribute to altered physiological responses and performance outcomes. The model integrates evidence from human and experimental studies, and some pathways remain hypothetical. Legend: Solid arrows indicate relationships supported by human evidence. Dotted arrows denote hypothesised or emerging pathways with limited empirical support. Bidirectional arrows indicate potential feedback interactions between systems. Abbreviations: SCFAs—short-chain fatty acids; BCAAs—branched-chain amino acids; AMPK—AMP-activated protein kinase; PPARγ—peroxisome proliferator-activated receptor gamma; ARG—antimicrobial resistance genes; Akt—protein kinase B.

To provide an integrated overview, Figure 1 presents a conceptual model of the interactions between travel-related stressors, gut microbiota alterations, and their potential physiological consequences in athletes.

### 6.4. Challenges and Gaps in Microbiota-Performance Research

Despite promising results, the available evidence on the role of GM in athletic performance has several limitations. Human studies are based on relatively small sample sizes and homogeneous populations, often limited to male endurance athletes, which restricts the generalizability of the findings. In addition, intervention periods are typically short, making it difficult to assess the long-term sustainability of the observed performance benefits. Interindividual variability in diet, training load, and baseline microbiota composition is not fully controlled and may influence responsiveness to probiotic supplementation.

Although animal studies provide important mechanistic insights, their translational relevance is limited due to fundamental physiological and metabolic differences between rodents and humans. Germ-free and microbiota-depleted mouse models represent extreme conditions that may overestimate the role of microbiota in muscle mass and performance regulation. Furthermore, probiotic doses and controlled experimental conditions in animal studies do not fully reflect real-world human settings. Collectively, these limitations highlight the need for larger, long-term human studies involving diverse populations and integrated mechanistic approaches. Future research should aim to better integrate microbiome, metabolic, and performance outcomes to clarify the role of GM in athletic contexts.

## 7. Strategies to Mitigate Travel-Induced Circadian and Microbiota Disruption

Table 1 summarises practical strategies that may help mitigate the effects of travel-induced circadian disruption and GM disturbances. Rather than relying on a single intervention, these approaches can be viewed as complementary strategies targeting interconnected domains, including circadian realignment, sleep support, gastrointestinal function, hydration, and dietary modulation. In this context, interventions such as appropriately timed light exposure, melatonin use, meal timing (chrononutrition), sleep hygiene, and nutritional support may contribute to post-travel adaptation.

However, these strategies should be interpreted as evidence-informed considerations rather than formal recommendations. Although some approaches are supported by broader chronobiology and sports science literature, evidence for several microbiota-focused interventions remains limited, particularly in athlete populations. Consequently, their implementation should be individualised and tailored to the athlete’s travel itinerary, symptom profile, dietary practices, and recovery needs.

## 8. Conclusions

Circadian rhythm disruption and GM dysregulation are closely interconnected and may influence human physiology, particularly in athletes exposed to frequent travel, stress, and time zone shifts. These disruptions have been associated with alterations in metabolism, immune function, cognitive performance, and recovery capacity. Evidence shows that travel-induced changes in sleep, stress, and environment lead to measurable shifts in microbial diversity and function, which may influence health and performance outcomes. Although important challenges persist—particularly the large differences between individuals and the difficulty of predicting environmental influences—advances in our understanding of circadian biology and microbiota-related processes may support the development of more targeted and individualized interventions.

This challenge is particularly relevant given the increasing intensity of the international sports calendar, with athletes travelling more frequently than ever. A prime example is the upcoming 2026 FIFA World Cup, which will be hosted across six time zones in North America. Such extreme logistical demands expose players to reported trans-meridian travel, circadian misalignment, and environmental transitions—factors that have been associated with reduced physiological resilience and an increased risk of underperformance.

As evidence accumulates, targeted strategies—such as timed light exposure, chrononutrition, and microbiota-supportive approaches (including probiotics and prebiotics)—have been proposed as potential strategies to mitigate these effects. Building personalized, evidence-based protocols that integrate circadian and microbiota health may contribute to optimising athlete preparation in high-stakes competitions. Future research should focus on bridging mechanistic understanding with practical applications to better prepare athletes for the physiological demands of global events. Understanding how the microbiota interacts with the body’s internal clock may open new avenues for research into recovery, immunity, and more personalised approaches to athletic training.

## Figures and Tables

**Table 1:** Potential strategies addressing travel-related circadian and gut microbiota disruptions in athletes.

Challenge	Mechanism	Intervention	Practical Implementation	References
Circadian misalignment (jet lag)	SCN desynchronization due to time zone shift	Timed light exposure	Light exposure strategies, aligned with circadian phase (e.g., relative to CBTmin), may support post-travel circadian realignment. This may include increasing or limiting exposure to bright light at specific times of day, as well as the use of environmental or behavioural measures (e.g., light avoidance or controlled light exposure).	[120,121,122]
		Melatonin supplementation	Melatonin has been proposed as a strategy to support circadian realignment following long-distance travel, particularly after eastward flights. Its timing may be adjusted relative to individual circadian phase (e.g., CBTmin), although optimal protocols in athletes remain to be established.	[123]
		Chronobiotic meal timing	Alignment of meal timing with the destination time zone (chrononutrition) has been proposed as a strategy to support circadian adaptation. This may include shifting feeding patterns toward daytime hours at the destination and avoiding food intake during the biological night.	[121,124]
Gut microbiota dysbiosis	SCFA production loss, reduced microbial diversity	Probiotic supplementation	Microbiota-targeted interventions, including probiotics and prebiotics, have been proposed as potential strategies to support gut microbiota function. Their effects may depend on dietary context, particularly the availability of fermentable substrates such as dietary fibre.	[125,126,127,128]
		Prebiotic fibre intake	Prebiotics have been proposed as a strategy to support gut microbiota composition and SCFA production. Their effects may depend on baseline dietary patterns, including habitual fibre intake and initial microbiota characteristics.	[128,129,130,131,132]
Sleep disturbance Gi disturbances (constipation, diarrhoea)	Circadian desync, stress, unfamiliar environment	Sleep hygiene practices	Strategies targeting circadian regulation of cortisol have been proposed, including management of light exposure, timing of physical activity, and sleep-related behaviours. Approaches such as reducing evening stimulation and supporting morning light exposure may help align circadian hormonal rhythms.	[24,32,34,40,45,46,64,65]
		Consistent sleep–wake schedule	Sleep hygiene-related strategies have been proposed to support sleep quality and circadian stability, including optimisation of the sleep environment and behavioural routines. Factors such as light exposure, dietary habits, and pre-sleep behaviours may influence sleep continuity and overall recovery.	[57,64,66]
	Slowed motility, altered microbiota, and dehydration	Hydration and electrolyte support	Adequate hydration has been proposed as an important factor supporting physiological function, gastrointestinal integrity, and recovery. Hydration needs may vary depending on exercise intensity, environmental conditions, and individual characteristics.	[59,133]
Increased stress & cortisol		SCFA-enhancing diet	Dietary fibre intake has been proposed as an important factor supporting gut microbiota diversity and metabolic function. Increasing fibre intake, including sources of resistant starch and prebiotics, may influence microbiota composition and short-chain fatty acid production.	[95,114,125,129,130,131,134,135,136]
	HPA axis activation, gut barrier impairment	Reduction in pro-inflammatory and HPA-axis-activating signalling	Probiotic supplementation has been proposed as a potential strategy to support stress-related outcomes and gut–brain axis interactions, although evidence in athletic populations remains limited. Exposure to natural environments may also be associated with beneficial effects on stress and overall well-being.	[132]

## Data Availability

No new data were created or analyzed in this study. Data sharing is not applicable to this article.

## References

[1] Dallmann R., Brown S.A., Gachon F. (2014). Chronopharmacology: New Insights and Therapeutic Implications. Annu. Rev. Pharmacol. Toxicol..

[2] Cedernaes J., Schönke M., Westholm J.O., Mi J., Chibalin A., Voisin S., Osler M., Vogel H., Hörnaeus K., Dickson S.L. (2018). Acute Sleep Loss Results in Tissue-Specific Alterations in Genome-Wide DNA Methylation State and Metabolic Fuel Utilization in Humans. Sci. Adv..

[3] Voigt R.M., Forsyth C.B., Green S.J., Mutlu E., Engen P., Vitaterna M.H., Turek F.W., Keshavarzian A. (2014). Circadian Disorganization Alters Intestinal Microbiota. PLoS ONE.

[4] Chodowiec A., Tarasewicz M., Łokić A., Kazberuk M., Panasiuk A. (2024). Biological Rhythms of the Gut and Microbiota. Prz. Gastroenterol..

[5] Lopez-Santamarina A., Mondragon A.d.C., Cardelle-Cobas A., Santos E.M., Porto-Arias J.J., Cepeda A., Miranda J.M. (2023). Effects of Unconventional Work and Shift Work on the Human Gut Microbiota and the Potential of Probiotics to Restore Dysbiosis. Nutrients.

[6] Shalmon G., Ibrahim R., Israel-Elgali I., Grad M., Shlayem R., Shapira G., Shomron N., Youngster I., Scheinowitz M. (2024). Gut Microbiota Composition Positively Correlates with Sports Performance in Competitive Non-Professional Female and Male Runners. Life.

[7] Li J., Yu K., Sui X., Deng H., Leng Y., Liu T. (2025). Gut Jet Lag: How Circadian Rhythm Disruption Undermines the Chrono-Microbiota-Motility Axis and Induces Functional Constipation. Front. Nutr..

[8] Mach N., Fuster-Botella D. (2017). Endurance Exercise and Gut Microbiota: A Review. J. Sport Health Sci..

[9] Samuels C.H. (2012). Jet Lag and Travel Fatigue: A Comprehensive Management Plan for Sport Medicine Physicians and High-Performance Support Teams. Clin. J. Sport Med..

[10] Lo M., Aughey R.J., Hopkins W.G., Gill N., Stewart A.M. (2019). The Longest Journeys in Super Rugby: 11 Years of Travel and Performance Indicators. J. Sports Sci..

[11] Fullagar H.H.K., Duffield R., Skorski S., Coutts A.J., Julian R., Meyer T. (2015). Sleep and Recovery in Team Sport: Current Sleep-Related Issues Facing Professional Team-Sport Athletes. Int. J. Sports Physiol. Perform..

[12] Halson S.L., Burke L.M., Pearce J. (2019). Nutrition for Travel: From Jet Lag To Catering. Int. J. Sport Nutr. Exerc. Metab..

[13] Rossiter M. (2023). Health Risks to Athletes at Olympic and Commonwealth Games. Occup. Med..

[14] Moore R.Y., Eichler V.B. (1972). Loss of a Circadian Adrenal Corticosterone Rhythm Following Suprachiasmatic Lesions in the Rat. Brain Res..

[15] Ralph M.R., Foster R.G., Davis F.C., Menaker M. (1990). Transplanted Suprachiasmatic Nucleus Determines Circadian Period. Science.

[16] Todd W.D., Venner A., Anaclet C., Broadhurst R.Y., De Luca R., Bandaru S.S., Issokson L., Hablitz L.M., Cravetchi O., Arrigoni E. (2020). Suprachiasmatic VIP Neurons Are Required for Normal Circadian Rhythmicity and Comprised of Molecularly Distinct Subpopulations. Nat. Commun..

[17] Mohawk J.A., Takahashi J.S. (2011). Cell Autonomy and Synchrony of Suprachiasmatic Nucleus Circadian Oscillators. Trends Neurosci..

[18] Bernard S., Gonze D., Čajavec B., Herzel H., Kramer A. (2007). Synchronization-Induced Rhythmicity of Circadian Oscillators in the Suprachiasmatic Nucleus. PLoS Comput. Biol..

[19] Ramkisoensing A., Meijer J.H. (2015). Synchronization of Biological Clock Neurons by Light and Peripheral Feedback Systems Promotes Circadian Rhythms and Health. Front. Neurol..

[20] Lucas R.J., Peirson S.N., Berson D.M., Brown T.M., Cooper H.M., Czeisler C.A., Figueiro M.G., Gamlin P.D., Lockley S.W., O’Hagan J.B. (2014). Measuring and Using Light in the Melanopsin Age. Trends Neurosci..

[21] Kalsbeek A., Palm I.F., La Fleur S.E., Scheer F.A.J.L., Perreau-Lenz S., Ruiter M., Kreier F., Cailotto C., Buijs R.M. (2006). SCN Outputs and the Hypothalamic Balance of Life. J. Biol. Rhythm..

[22] Khalsa S.B.S., Jewett M.E., Cajochen C., Czeisler C.A. (2003). A Phase Response Curve to Single Bright Light Pulses in Human Subjects. J. Physiol..

[23] Czeisler C.A., Duffy J.F., Shanahan T.L., Brown E.N., Mitchell J.F., Rimmer D.W., Ronda J.M., Silva E.J., Allan J.S., Emens J.S. (1999). Stability, Precision, and near-24-Hour Period of the Human Circadian Pacemaker. Science.

[24] Doherty R., Madigan S.M., Nevill A., Warrington G., Ellis J.G. (2023). The Impact of Long Haul Travel on the Sleep of Elite Athletes. Neurobiol. Sleep Circadian Rhythm..

[25] Biggins M., Purtill H., Fowler P., O’Sullivan K., Cahalan R. (2022). Impact of Long-Haul Travel to International Competition on Sleep and Recovery in Elite Male and Female Soccer Athletes. Int. J. Sports Physiol. Perform..

[26] Youngstedt S.D., Elliott J.A., Kripke D.F. (2019). Human Circadian Phase–Response Curves for Exercise. J. Physiol..

[27] Hughes A.T.L. (2018). Locomotor Exercise and Circadian Rhythms in Mammals. Curr. Opin. Physiol..

[28] Thomas J.M., Kern P.A., Bush H.M., McQuerry K.J., Black W.S., Clasey J.L., Pendergast J.S. (2020). Circadian Rhythm Phase Shifts Caused by Timed Exercise Vary with Chronotype. JCI Insight.

[29] Damiola F., Le Minli N., Preitner N., Kornmann B., Fleury-Olela F., Schibler U. (2000). Restricted Feeding Uncouples Circadian Oscillators in Peripheral Tissues from the Central Pacemaker in the Suprachiasmatic Nucleus. Genes Dev..

[30] Burke T.M., Markwald R.R., McHill A.W., Chinoy E.D., Snider J.A., Bessman S.C., Jung C.M., O’Neill J.S., Wright K.P. (2015). Effects of Caffeine on the Human Circadian Clock In Vivo and In Vitro. Sci. Transl. Med..

[31] Cho K., Ennaceur A., Cole J.C., Suh C.K. (2000). Chronic Jet Lag Produces Cognitive Deficits. J. Neurosci..

[32] Paragliola R.M., Corsello A., Troiani E., Locantore P., Papi G., Donnini G., Pontecorvi A., Corsello S.M., Carrozza C. (2021). Cortisol Circadian Rhythm and Jet-Lag Syndrome: Evaluation of Salivary Cortisol Rhythm in a Group of Eastward Travelers. Endocrine.

[33] Gi H.S., Chung S., Han K.C., Kim H.D., Baik S.M., Lee H., Lee H.W., Choi S., Sun W., Kim H. (2008). Adrenal Peripheral Clock Controls the Autonomous Circadian Rhythm of Glucocorticoid by Causing Rhythmic Steroid Production. Proc. Natl. Acad. Sci. USA.

[34] Leproult R., Holmbäck U., Van Cauter E. (2014). Circadian Misalignment Augments Markers of Insulin Resistance and Inflammation, Independently of Sleep Loss. Diabetes.

[35] Scheer F.A.J.L., Hilton M.F., Mantzoros C.S., Shea S.A. (2009). Adverse Metabolic and Cardiovascular Consequences of Circadian Misalignment. Proc. Natl. Acad. Sci. USA.

[36] Adams K.L., Castanon-Cervantes O., Evans J.A., Davidson A.J. (2013). Environmental Circadian Disruption Elevates the IL-6 Response to Lipopolysaccharide in Blood. J. Biol. Rhythm..

[37] Van Mark A., Weiler S.W., Schröder M., Otto A., Jauch-Chara K., Groneberg D.A., Spallek M., Kessel R., Kalsdorf B. (2010). The Impact of Shift Work Induced Chronic Circadian Disruption on IL-6 and TNF-Alpha Immune Responses. J. Occup. Med. Toxicol..

[38] Thorkildsen M.S., Gustad L.T., Damås J.K. (2023). The Effects of Shift Work on the Immune System: A Narrative Review. Sleep Sci..

[39] Manfredini R., Manfredini F., Fersini C., Francesco C. (1998). Circadian Rhythms, Athletic Performance, and Jet Lag. Br. J. Sports Med..

[40] Charest J., Grandner M.A. (2020). Sleep and Athletic Performance: Impacts on Physical Performance, Mental Performance, Injury Risk and Recovery, and Mental Health. Sleep Med. Clin..

[41] Kalmbach D.A., Schneider L.D., Cheung J., Bertrand S.J., Kariharan T., Pack A.I., Gehrman P.R. (2016). Genetic Basis of Chronotype in Humans: Insights From Three Landmark GWAS. Sleep.

[42] Facer-Childs E.R., Boiling S., Balanos G.M. (2018). The Effects of Time of Day and Chronotype on Cognitive and Physical Performance in Healthy Volunteers. Sports Med. Open.

[43] Lok R., Zerbini G., Gordijn M.C.M., Beersma D.G.M., Hut R.A. (2020). Gold, Silver or Bronze: Circadian Variation Strongly Affects Performance in Olympic Athletes. Sci. Rep..

[44] Leota J., Hoffman D., Czeisler M., Mascaro L., Drummond S.P.A., Anderson C., Rajaratnam S.M.W., Facer-Childs E.R. (2022). Eastward Jet Lag Is Associated with Impaired Performance and Game Outcome in the National Basketball Association. Front. Physiol..

[45] Song A., Severini T., Allada R. (2017). How Jet Lag Impairs Major League Baseball Performance. Proc. Natl. Acad. Sci. USA.

[46] Thaiss C.A., Zeevi D., Levy M., Zilberman-Schapira G., Suez J., Tengeler A.C., Abramson L., Katz M.N., Korem T., Zmora N. (2014). Transkingdom Control of Microbiota Diurnal Oscillations Promotes Metabolic Homeostasis. Cell.

[47] Leone V., Gibbons S.M., Martinez K., Hutchison A.L., Huang E.Y., Cham C.M., Pierre J.F., Heneghan A.F., Nadimpalli A., Hubert N. (2015). Effects of Diurnal Variation of Gut Microbes and High-Fat Feeding on Host Circadian Clock Function and Metabolism. Cell Host Microbe.

[48] Segers A., Depoortere I. (2021). Circadian Clocks in the Digestive System. Nat. Rev. Gastroenterol. Hepatol..

[49] Frazier K., Kambal A., Zale E.A., Pierre J.F., Hubert N., Miyoshi S., Miyoshi J., Ringus D.L., Harris D., Yang K. (2022). High-fat diet disrupts REG3γ and gut microbial rhythms promoting metabolic dysfunction. Cell Host Microbe.

[50] Ma J., Zhang J., Kuang Z. (2023). A Microbiota-Epigenetic Circuit Controls Systematic Circadian Programs in the Gut Epithelium. Front. Syst. Biol..

[51] Govindarajan K., MacSharry J., Casey P.G., Shanahan F., Joyce S.A., Gahan C.G.M. (2016). Unconjugated Bile Acids Influence Expression of Circadian Genes: A Potential Mechanism for Microbe-Host Crosstalk. PLoS ONE.

[52] Niu Y., Heddes M., Altaha B., Birkner M., Kleigrewe K., Meng C., Haller D., Kiessling S. (2024). Targeting the Intestinal Circadian Clock by Meal Timing Ameliorates Gastrointestinal Inflammation. Cell. Mol. Immunol..

[53] Cheng L., Wang X., Wang Q., Yin K., Wang B., Wu B., Xu P., Qiu H., Ge W., Sun J. (2026). Circadian Rhythm Disturbance Impairs Intestinal Mucus Barrier and Immune Microenvironment via Sebacic Acid-Mediated Gut Dysbiosis. Microbiol. Res..

[54] Ahmad S.R., AlShahrani A.M., Kumari A. (2025). Effects of Probiotic Supplementation on Depressive Symptoms, Sleep Quality, and Modulation of Gut Microbiota and Inflammatory Biomarkers: A Randomized Controlled Trial. Brain Sci..

[55] Chen I.Y., Radom-Aizik S., Stehli A., Palmer J.R., Lui K.K., Dave A., Chappel-Farley M.G., Vinces K.G., Gealer D., Lim A. (2024). Cardiorespiratory Fitness and Circadian Rhythms in Adolescents: A Pilot Study. J. Appl. Physiol..

[56] Wang W., Chen H. (2024). A Systematic Review and Meta-Analysis Study on the Factors Affecting Sports Psychology, Athletic Performance and Physical Activity. Med. Dello Sport.

[57] Doherty R., Madigan S., Warrington G., Ellis J.G. (2023). Sleep and Nutrition in Athletes. Curr. Sleep Med. Rep..

[58] De Zan D., Eletti F., Fiore G., Di Girolamo E., Bozzini G.G.M., Perico V., Tosi M., Norsa L., Zuccotti G., Verduci E. (2025). Use of Nutritional Strategies, Bioactive Compounds, and Dietary Supplements in Young Athletes: From Evidence to Potential Risks—A Narrative Review. Nutrients.

[59] Papaoikonomou G., Kandyliari A., Vlassopoulos A., Malisova O., Koutelidakis A.E. (2025). Hydration Status, Dietary Habits, and Functional Food Consumption Preferences of Football Athletes: A Cross-Sectional Pilot Study. Nutrients.

[60] Bongiovanni T., Santiago M., Zielinska K., Scheiman J., Barsa C., Jäger R., Pinto D., Rinaldi F., Giuliani G., Senatore T. (2025). A Lactobacillus Consortium Provides Insights into the Sleep-Exercise-Microbiome Nexus in Proof-of-Concept Studies of Elite Athletes and in the General Population. Microbiome.

[61] Bermingham K.M., Stensrud S., Asnicar F., Valdes A.M., Franks P.W., Wolf J., Hadjigeorgiou G., Davies R., Spector T.D., Segata N. (2023). Exploring the Relationship between Social Jetlag with Gut Microbial Composition, Diet and Cardiometabolic Health, in the ZOE PREDICT 1 Cohort. Eur. J. Nutr..

[62] Shaidullov I.F., Sorokina D.M., Sitdikov F.G., Hermann A., Abdulkhakov S.R., Sitdikova G.F. (2021). Short Chain Fatty Acids and Colon Motility in a Mouse Model of Irritable Bowel Syndrome. BMC Gastroenterol..

[63] Zhu M.T., Lee J.W.J. (2025). Therapeutic Potential of Short-Chain Fatty Acids in Gastrointestinal Diseases. Nutraceuticals.

[64] Ampofo J., Sun B., Bentum-Micah G., Qinggong L., Changfeng W., Guoan L., Xusheng Q. (2025). Investigating the Impact of Sleep Quality on Cognitive Functions among Students in Tokyo, Japan, and London, UK. Front. Sleep.

[65] Kudrnáčová M., Kudrnáč A. (2023). Better Sleep, Better Life? Testing the Role of Sleep on Quality of Life. PLoS ONE.

[66] Tadros M., Newby J.M., Li S., Werner-Seidler A. (2025). A Systematic Review and Meta-Analysis of Psychological Treatments to Improve Sleep Quality in University Students. PLoS ONE.

[67] Smith R.P., Easson C., Lyle S.M., Kapoor R., Donnelly C.P., Davidson E.J., Parikh E., Lopez J.V., Tartar J.L. (2019). Gut Microbiome Diversity Is Associated with Sleep Physiology in Humans. PLoS ONE.

[68] Li C., Yao J., Yang C., Yu S., Yang Z., Wang L., Li S., He N. (2025). Gut Microbiota-Derived Short-Chain Fatty Acids Act as Mediators of the Gut-Liver-Brain Axis. Metab. Brain Dis..

[69] Mukhopadhya I., Louis P. (2025). Gut Microbiota-Derived Short-Chain Fatty Acids and Their Role in Human Health and Disease. Nat. Rev. Microbiol..

[70] Chulenbayeva L., Issilbayeva A., Sailybayeva A., Bekbossynova M., Kozhakhmetov S., Kushugulova A. (2025). Short-Chain Fatty Acids and Their Metabolic Interactions in Heart Failure. Biomedicines.

[71] Collins S.L., Stine J.G., Bisanz J.E., Okafor C.D., Patterson A.D. (2023). Bile Acids and the Gut Microbiota: Metabolic Interactions and Impacts on Disease. Nat. Rev. Microbiol..

[72] Su X., Gao Y., Yang R. (2023). Gut Microbiota Derived Bile Acid Metabolites Maintain the Homeostasis of Gut and Systemic Immunity. Front. Immunol..

[73] Rusch J.A., Layden B.T., Dugas L.R. (2023). Signalling Cognition: The Gut Microbiota and Hypothalamic-Pituitary-Adrenal Axis. Front. Endocrinol..

[74] Yu L.E., Yang W.C., Liang Y.C. (2024). Crosstalk Within the Intestinal Epithelium: Aspects of Intestinal Absorption, Homeostasis, and Immunity. Biomedicines.

[75] Shibata C., Muratsubaki T., Shibata S., Aizawa E., Watanabe S., Kanazawa M., Fukudo S. (2025). A Randomized Controlled Trial of Environmental Richness on Gastrointestinal Symptoms, Salivary Cortisol, and Gut Microbiota in Early Childhood. Sci. Rep..

[76] Wauters L., Ceulemans M., Schol J., Farré R., Tack J., Vanuytsel T. (2022). The Role of Leaky Gut in Functional Dyspepsia. Front. Neurosci..

[77] Vanuytsel T., Van Wanrooy S., Vanheel H., Vanormelingen C., Verschueren S., Houben E., Rasoel S.S., Tóth J., Holvoet L., Farré R. (2014). Psychological Stress and Corticotropin-Releasing Hormone Increase Intestinal Permeability in Humans by a Mast Cell-Dependent Mechanism. Gut.

[78] Dmytriv T.R., Storey K.B., Lushchak V.I. (2024). Intestinal Barrier Permeability: The Influence of Gut Microbiota, Nutrition, and Exercise. Front. Physiol..

[79] Jain M., Anand A., Sharma N., Shamim M.A., Enioutina E.Y. (2024). Effect of Probiotics Supplementation on Cortisol Levels: A Systematic Review and Meta-Analysis. Nutrients.

[80] Sivri D., Seref B., Sare Bulut M., Gezmen Karadaǧ M. (2025). Evaluation of the Effect of Probiotic Supplementation on Intestinal Barrier Integrity and Epithelial Damage in Colitis Disease: A Systematic Review. Nutr. Rev..

[81] Safabakhsh M., Shab-Bidar S., Rohani P., Beirami F., Mohammadpour M., Imani H. (2025). Effect of Probiotics on Intestinal Permeability in Critically Ill Children with Sepsis: Preliminary Results from a Double-Blind, Placebo-Controlled Trial. Eur. J. Nutr..

[82] Francisco Vázquez-Castellanos J., Ferreira Maciel L., Wauters L., Gregory A., Van Oudenhove L., Geboers K., Verbeke K., Smokvina T., Tack J., Vanuytsel T. (2025). Probiotic-Mediated Modulation of Gut Microbiome in Students Exposed to Academic Stress: A Randomized Controlled Trial. npj Biofilms Microbiomes.

[83] Jarrett H., Medlin S., Morehen J.C. (2025). The Role of the Gut Microbiome and Probiotics in Sports Performance: A Narrative Review Update. Nutrients.

[84] Shi Y., Wang P., Zhou D., Huang L., Zhang L., Gao X., Maitiabula G., Wang S., Wang X. (2022). Multi-Omics Analyses Characterize the Gut Microbiome and Metabolome Signatures of Soldiers Under Sustained Military Training. Front. Microbiol..

[85] Mancin L., Paoli A., Berry S., Gonzalez J.T., Collins A.J., Lizarraga M.A., Mota J.F., Nicola S., Rollo I. (2024). Standardization of Gut Microbiome Analysis in Sports. Cell Rep. Med..

[86] Schwingshackl L., Balduzzi S., Beyerbach J., Bröckelmann N., Werner S.S., Zähringer J., Nagavci B., Meerpohl J.J. (2021). Evaluating Agreement between Bodies of Evidence from Randomised Controlled Trials and Cohort Studies in Nutrition Research: Meta-Epidemiological Study. BMJ.

[87] Tobias D.K., Lajous M. (2021). What Would the Trial Be? Emulating Randomized Dietary Intervention Trials to Estimate Causal Effects with Observational Data. Am. J. Clin. Nutr..

[88] Ross F.C., Patangia D., Grimaud G., Lavelle A., Dempsey E.M., Ross R.P., Stanton C. (2024). The Interplay between Diet and the Gut Microbiome: Implications for Health and Disease. Nat. Rev. Microbiol..

[89] Minoretti P., Sigurtà C., Fachinetti A., Cerone E., Rotta F., Emanuele E. (2023). A Preliminary Study of Gut Microbiota in Airline Pilots: Comparison with Construction Workers and Fitness Instructors. Cureus.

[90] Effendi R.M.R.A., Anshory M., Kalim H., Dwiyana R.F., Suwarsa O., Pardo L.M., Nijsten T.E.C., Thio H.B. (2022). *Akkermansia muciniphila* and *Faecalibacterium prausnitzii* in Immune-Related Diseases. Microorganisms.

[91] Lopez-Siles M., Enrich-Capó N., Aldeguer X., Sabat-Mir M., Duncan S.H., Garcia-Gil L.J., Martinez-Medina M. (2018). Alterations in the Abundance and Co-Occurrence of *Akkermansia muciniphila* and *Faecalibacterium prausnitzii* in the Colonic Mucosa of Inflammatory Bowel Disease Subjects. Front. Cell. Infect. Microbiol..

[92] Han E.J., Ahn J.S., Chae Y.J., Chung H.J. (2025). Immunomodulatory Roles of *Faecalibacterium prausnitzii* and *Akkermansia muciniphila* in Autoimmune Diseases: Mechanistic Insights and Therapeutic Potential. Clin. Rev. Allergy Immunol..

[93] Worby C.J., Sridhar S., Turbett S.E., Becker M.V., Kogut L., Sanchez V., Bronson R.A., Rao S.R., Oliver E., Walker A.T. (2023). Gut Microbiome Perturbation, Antibiotic Resistance, and *Escherichia coli* Strain Dynamics Associated with International Travel: A Metagenomic Analysis. Lancet Microbe.

[94] Martín R., Rios-Covian D., Huillet E., Auger S., Khazaal S., Bermúdez-Humarán L.G., Sokol H., Chatel J.M., Langella P. (2023). *Faecalibacterium*: A Bacterial Genus with Promising Human Health Applications. FEMS Microbiol. Rev..

[95] Kim Y.J., Jung D.H., Park C.S. (2024). Important Roles of Ruminococcaceae in the Human Intestine for Resistant Starch Utilization. Food Sci. Biotechnol..

[96] Nishiyama K., Yokoi T., Sugiyama M., Osawa R., Mukai T., Okada N. (2021). Roles of the Cell Surface Architecture of *Bacteroides* and *Bifidobacterium* in the Gut Colonization. Front. Microbiol..

[97] Fuhrmeister E.R., Harvey A.P., Nadimpalli M.L., Gallandat K., Ambelu A., Arnold B.F., Brown J., Cumming O., Earl A.M., Kang G. (2023). Evaluating the Relationship between Community Water and Sanitation Access and the Global Burden of Antibiotic Resistance: An Ecological Study. Lancet Microbe.

[98] Lefevre C., Bindels L.B. (2022). Role of the Gut Microbiome in Skeletal Muscle Physiology and Pathophysiology. Curr. Osteoporos. Rep..

[99] Chen Y.M., Wei L., Chiu Y.S., Hsu Y.J., Tsai T.Y., Wang M.F., Huang C.C. (2016). *Lactobacillus plantarum* TWK10 Supplementation Improves Exercise Performance and Increases Muscle Mass in Mice. Nutrients.

[100] Bressa C., Bailén-Andrino M., Pérez-Santiago J., González-Soltero R., Pérez M., Montalvo-Lominchar M.G., Maté-Muñoz J.L., Domínguez R., Moreno D., Larrosa M. (2017). Differences in Gut Microbiota Profile between Women with Active Lifestyle and Sedentary Women. PLoS ONE.

[101] Nay K., Jollet M., Goustard B., Baati N., Vernus B., Pontones M., Lefeuvre-Orfila L., Bendavid C., Rué O., Mariadassou M. (2019). Gut Bacteria Are Critical for Optimal Muscle Function: A Potential Link with Glucose Homeostasis. Am. J. Physiol. Endocrinol. Metab..

[102] Lahiri S., Kim H., Garcia-Perez I., Reza M.M., Martin K.A., Kundu P., Cox L.M., Selkrig J., Posma J.M., Zhang H. (2019). The Gut Microbiota Influences Skeletal Muscle Mass and Function in Mice. Sci. Transl. Med..

[103] Scheiman J., Luber J.M., Chavkin T.A., MacDonald T., Tung A., Pham L.D., Wibowo M.C., Wurth R.C., Punthambaker S., Tierney B.T. (2019). Meta-Omics Analysis of Elite Athletes Identifies a Performance-Enhancing Microbe That Functions via Lactate Metabolism. Nat. Med..

[104] Ghorbani Asiabar M., Ghorbani Asiabar A. (2023). The Impact of Gut Microbiome Modulation on Athletic Performance and Post-Exercise Recovery in Endurance Runners. New Approaches Exerc. Physiol..

[105] Munukka E., Ahtiainen J.P., Puigbó P., Jalkanen S., Pahkala K., Keskitalo A., Kujala U.M., Pietilä S., Hollmén M., Elo L. (2018). Six-Week Endurance Exercise Alters Gut Metagenome That Is Not Reflected in Systemic Metabolism in Overweight Women. Front. Microbiol..

[106] Estaki M., Pither J., Baumeister P., Little J.P., Gill S.K., Ghosh S., Ahmadi-Vand Z., Marsden K.R., Gibson D.L. (2016). Cardiorespiratory Fitness as a Predictor of Intestinal Microbial Diversity and Distinct Metagenomic Functions. Microbiome.

[107] Bycura D., Santos A.C., Shiffer A., Kyman S., Winfree K., Sutliffe J., Pearson T., Sonderegger D., Cope E., Caporaso J.G. (2021). Impact of Different Exercise Modalities on the Human Gut Microbiome. Sports.

[108] O’Donovan C.M., Madigan S.M., Garcia-Perez I., Rankin A., O’Sullivan O., Cotter P.D. (2020). Distinct Microbiome Composition and Metabolome Exists across Subgroups of Elite Irish Athletes. J. Sci. Med. Sport.

[109] Barton W., Penney N.C., Cronin O., Garcia-Perez I., Molloy M.G., Holmes E., Shanahan F., Cotter P.D., O’Sullivan O. (2018). The Microbiome of Professional Athletes Differs from That of More Sedentary Subjects in Composition and Particularly at the Functional Metabolic Level. Gut.

[110] Martin D., Bonneau M., Orfila L., Horeau M., Hazon M., Demay R., Lecommandeur E., Boumpoutou R., Guillotel A., Guillemot P. (2025). Atypical Gut Microbial Ecosystem from Athletes with Very High Exercise Capacity Improves Insulin Sensitivity and Muscle Glycogen Store in Mice. Cell Rep..

[111] Sales K.M., Reimer R.A. (2023). Unlocking a Novel Determinant of Athletic Performance: The Role of the Gut Microbiota, Short-Chain Fatty Acids, and “Biotics” in Exercise. J. Sport Health Sci..

[112] Xu Y., He B. (2025). The Gut-Muscle Axis: A Comprehensive Review of the Interplay between Physical Activity and Gut Microbiota in the Prevention and Treatment of Muscle Wasting Disorders. Front. Microbiol..

[113] Bertocci L.A., Jones J.G., Malloy C.R., Victor R.G., Thomas G.D. (1997). Oxidation of Lactate and Acetate in Rat Skeletal Muscle: Analysis by 13C-Nuclear Magnetic Resonance Spectroscopy. J. Appl. Physiol..

[114] Okamoto T., Morino K., Ugi S., Nakagawa F., Lemecha M., Ida S., Ohashi N., Sato D., Fujita Y., Maegawa H. (2019). Microbiome Potentiates Endurance Exercise through Intestinal Acetate Production. Am. J. Physiol. Endocrinol. Metab..

[115] Maruta H., Yoshimura Y., Araki A., Kimoto M., Takahashi Y., Yamashita H. (2016). Activation of AMP-Activated Protein Kinase and Stimulation of Energy Metabolism by Acetic Acid in L6 Myotube Cells. PLoS ONE.

[116] Kjøbsted R., Hingst J.R., Fentz J., Foretz M., Sanz M.N., Pehmøller C., Shum M., Marette A., Mounier R., Treebak J.T. (2018). AMPK in Skeletal Muscle Function and Metabolism. FASEB J..

[117] Kim H.J., Kim Y.J., Kim Y.J., Baek J.H., Kim H.S., Kim I.Y., Seong J.K. (2023). Microbiota Influences Host Exercise Capacity via Modulation of Skeletal Muscle Glucose Metabolism in Mice. Exp. Mol. Med..

[118] Da Silva Rosa S.C., Nayak N., Caymo A.M., Gordon J.W. (2020). Mechanisms of Muscle Insulin Resistance and the Cross-Talk with Liver and Adipose Tissue. Physiol. Rep..

[119] Gao Z., Yin J., Zhang J., Ward R.E., Martin R.J., Lefevre M., Cefalu W.T., Ye J. (2009). Butyrate Improves Insulin Sensitivity and Increases Energy Expenditure in Mice. Diabetes.

[120] Roach G.D., Sargent C. (2019). Interventions to Minimize Jet Lag After Westward and Eastward Flight. Front. Physiol..

[121] Janse van Rensburg D.C., Jansen van Rensburg A., Fowler P.M., Bender A.M., Stevens D., O’Sullivan K., Fullagar H.H.K., Alonso J.M., Biggins M., Claassen-Smithers A. (2021). Managing Travel Fatigue and Jet Lag in Athletes: A Review and Consensus Statement. Sports Med..

[122] Serkh K., Forger D.B. (2014). Optimal Schedules of Light Exposure for Rapidly Correcting Circadian Misalignment. PLoS Comput. Biol..

[123] Herxheimer A., Petrie K.J. (2002). Melatonin for the Prevention and Treatment of Jet Lag. Cochrane Database Syst. Rev..

[124] Wehrens S.M.T., Christou S., Isherwood C., Middleton B., Gibbs M.A., Archer S.N., Skene D.J., Johnston J.D. (2017). Meal Timing Regulates the Human Circadian System. Curr. Biol..

[125] Son J., Jang L.G., Kim B.Y., Lee S., Park H. (2020). The Effect of Athletes’ Probiotic Intake May Depend on Protein and Dietary Fiber Intake. Nutrients.

[126] Brasiel P.G.D.A., Potente Dutra Luquetti S.C. (2025). Effects of Probiotics Supplementation on Short-Chain Fatty Acids: A Systematic Review of Randomized Controlled Trials. Nutr. Rev..

[127] Łagowska K., Bajerska J., Kamiński S., Del Bo’ C. (2022). Effects of Probiotics Supplementation on Gastrointestinal Symptoms in Athletes: A Systematic Review of Randomized Controlled Trials. Nutrients.

[128] Aparicio-Pascual D., Clemente-Suárez V.J., Tornero-Aguilera J.F., Rubio-Zarapuz A. (2025). The Effect of Probiotic Supplementation on Cytokine Modulation in Athletes After a Bout of Exercise: A Systematic Review and Meta-Analysis. Sports Med. Open.

[129] Czajkowska A., Szponar B. (2018). Short Chain Fatty Acids (SCFA), the Products of Gut Bacteria Metabolism and Their Role in the Host. Postep. Hig. Med. Dosw..

[130] Holmes Z.C., Villa M.M., Durand H.K., Jiang S., Dallow E.P., Petrone B.L., Silverman J.D., Lin P.H., David L.A. (2022). Microbiota Responses to Different Prebiotics Are Conserved within Individuals and Associated with Habitual Fiber Intake. Microbiome.

[131] Bai J., Li Y., Zhang W., Fan M., Qian H., Zhang H., Qi X., Wang L. (2021). Effects of Cereal Fibers on Short-Chain Fatty Acids in Healthy Subjects and Patients: A Meta-Analysis of Randomized Clinical Trials. Food Funct..

[132] Ahmadi-Khorram M., Hatami A., Asghari P., Jafarzadeh Esfehani A., Afshari A., Javdan F., Nematy M. (2025). Probiotics Mitigate Stress and Inflammation in Malnourished Adults via Gut Microbiota Modulation: A Randomized Controlled Trial. Front. Nutr..

[133] Rodriguez-Sanchez N., Galloway S.D.R. (2023). A Randomised Trial to Assess Fluid and Electrolyte Balance Responses Following Ingestion of Different Beverages in Young and Older Men. Eur. J. Appl. Physiol..

[134] Sobh M., Montroy J., Daham Z., Sibbald S., Lalu M., Stintzi A., Mack D., Fergusson D.A. (2022). Tolerability and SCFA Production after Resistant Starch Supplementation in Humans: A Systematic Review of Randomized Controlled Studies. Am. J. Clin. Nutr..

[135] Baxter N.T., Schmidt A.W., Venkataraman A., Kim K.S., Waldron C., Schmidt T.M. (2019). Dynamics of Human Gut Microbiota and Short-Chain Fatty Acids in Response to Dietary Interventions with Three Fermentable Fibers. mBio.

[136] Yang J., Martínez I., Walter J., Keshavarzian A., Rose D.J. (2013). In Vitro Characterization of the Impact of Selected Dietary Fibers on Fecal Microbiota Composition and Short Chain Fatty Acid Production. Anaerobe.

